# CKIP‐1 silencing suppresses OSCC via mitochondrial homeostasis‐associated TFAM/cGAS‐STING signalling axis

**DOI:** 10.1111/jcmm.70006

**Published:** 2024-08-21

**Authors:** Ji‐Rong Xie, Xiao‐Jie Chen, Gang Zhou

**Affiliations:** ^1^ State Key Laboratory of Oral and Maxillofacial Reconstruction and Regeneration, Key Laboratory of Oral Biomedicine Ministry of Education, Hubei Key Laboratory of Stomatology, School and Hospital of Stomatology Wuhan University Wuhan China; ^2^ Department of Oral Medicine, School and Hospital of Stomatology Wuhan University Wuhan China

**Keywords:** gene therapy, mitochondria, oral squamous cell carcinoma, signal transduction

## Abstract

Limited effective targets have challenged the treatment of oral squamous cell carcinoma (OSCC). Casein kinase 2 interacting protein 1 (CKIP‐1) is a scaffold protein involved in various diseases. However, the role of CKIP‐1 in OSCC remains unclear. The aim of this study was to explore the regulatory role of CKIP‐1 in OSCC, as well as the involved mechanism. First, higher expression of CKIP‐1 in OSCC tissues and cell lines were found. Series of gain‐ and loss‐of‐function experiments demonstrated suppressed malignant behaviours and enhanced apoptosis of OSCC cells when CKIP‐1 was silenced. Also, inhibited tumour growth in CKIP‐1‐silenced group were proved. Further, mitochondrial transcription factor A (TFAM) downregulation, increased ROS production, decreased mitochondrial membrane potential and cGAS‐STING activation in CKIP‐1‐silenced group were observed. The involvement of mitochondrial homeostasis‐related TFAM/cGAS‐STING axis in CKIP‐1‐silenced OSCC cells was finally demonstrated by tetramethylpyrazine (TMP) that inhibits TFAM degradation. Taken together, our study demonstrated that CKIP‐1 silencing could significantly antagonize OSCC via TFAM/cGAS‐STING axis, which may provide a candidate target for OSCC treatment.

## 1 INTRODUCTION

Head and neck squamous cell carcinoma (HNSCC) is the seventh most common malignancy around the world, accounting for 90% of all head and neck malignancies.^1^ Oral squamous cell carcinoma (OSCC) is the most common malignancy in HNSCC with high morbidity and mortality. Despite advances in diagnosis and treatment, the 5‐year survival rate of OSCC has no significant increase in recent years, with 50%–60% only.^2^, ^3^ The lack of improved clinical outcomes is thought to be associated with the limited availability of effective targeted therapies, calling for the exploration of new targets for OSCC treatment.^4^, ^5^

Casein kinase 2 interacting protein‐1 (CKIP‐1), also known as pleckstrin homology domain containing, family O member 1 (PLEKHO1), was initially identified to be a binding protein of CK2α.^6^ Gradually, it was found to interact with multiple proteins, influencing a variety of biological functions, such as osteogenesis, cementogenesis, immune regulation and tumorigenesis.^7^, ^8^, ^9^ So far, CKIP‐1 was reported to be associated with many tumours such as non‐Hodgkin's lymphoma, glioma, colonic and gastric cancer.^10^, ^11^, ^12^, ^13^, ^14^ However, the relationship between CKIP‐1 and OSCC remains unknown.

Mitochondrial transcription factor A (TFAM) is required for mitochondrial homeostasis and is involved in the metabolic changes that occur in tumours. It has been implicated to be a tumour‐promoter in hepatocellular carcinoma and breast cancer (BRCA).^15^, ^16^ Nevertheless, the regulatory effect of TFAM in OSCC has not been reported. Moreover, TFAM disruption‐induced mitochondrial dysfunction can activate the downstream cGAS‐STING pathway, which was proved to act as a tumour suppressor in OSCC.^17^, ^18^ Take these into consideration, the participation and function of TFAM/cGAS‐STING pathway in OSCC need further elucidation.

This study demonstrated the regulatory effect of CKIP‐1, and also the underlying mechanism in OSCC for the first time. Higher expression of CKIP‐1 was found in OSCC samples and cells. In vitro and in vivo experiments revealed that CKIP‐1 positively regulated the malignant behaviours of OSCC cells. In terms of mechanism, CKIP‐1 silencing induced TFAM downregulation disrupted mitochondrial homeostasis to activate the tumour‐suppressing cGAS‐STING axis in OSCC cells. Our study provides a potential candidate target for OSCC treatment.

## 2 MATERIALS AND METHODS

### 2.1 Patient cohorts and tumour tissues

One hundred and sixteen cases of OSCC samples (48 samples have paired paraneoplastic tissues) and 32 cases of normal oral mucosa samples were collected from Hospital of Stomatology, Wuhan University. This study was approved by the Ethics Committee of Wuhan University. Written informed consent was obtained from each participant.

### 2.2 Cell culture

The human OSCC cell lines SCC25, CAL27, HSC2 and SCC9 were obtained from the Cell Bank and Stem Cell Bank, Chinese Academy of Science and human immortalized oral epithelial cells (HOIECs) were established and obtained from School of Medicine, the Ninth People's Hospital, Shanghai Jiao Tong University. CAL27 and HSC2 were cultured in high glucose Dulbecco's modified eagle medium (DMEM; Gibco, CA, USA). SCC25 and SCC9 cells were cultured in DMEM: F12 (1: 1; Gibco, CA, USA). HOIECs were cultured in keratinocyte growth medium (KGM; Lonza, Basilea, Switzerland) 1% penicillin–streptomycin and 10% fetal bovine serum (FBS; BI, Kibbutz Beit Haemek, Israel) were added. Cells were cultured at 37°C in a humidified atmosphere containing 5% CO_2_.

### 2.3 TUNEL staining

3 × 10^4^ cells were seeded in 48‐well plates and fixed with 4% paraformaldehyde after 24 h. The TUNEL reagent was purchased from Elabscience (Wuhan, China). 100 μL of TdT Equilibration Buffer was added dropwise to each sample for 10–30 min at 37°C. The TdT Equilibration Buffer was removed by blotting paper. Each sample was then incubated with 50 μL of labeling solution for 60 min at 37°C in a wet box. The nuclei were stained by incubating with DAPI working solution (Beyotime, Nanjing, China) for 5 min at room temperature. After sealing, the cell or tissue samples were observed under a fluorescent microscope, and photographed for quantification.

### 2.4 Hoechst/PI double staining

1.2 × 10^5^ cells were seeded in the 24‐well plates and digested with trypsin 24 h later. Cells were digested and resuspended in 1 mL culture medium, and added with hoechst (Beyotime, Nanjing, China) with a final concentration of 16 μg/mL at 37° for 10 min. After centrifugation, 40 μg/mL PI (Beyotime, Nanjing, China) staining solution was added at 4°C for 15 min in the dark. The stained cell suspension was dropped on a slide and covered with a cover slip. Images were taken with a microscope, and quantified.

### 2.5 Flow cytometry

2.5 × 10^5^ cells were seeded in the 6‐well plates and were collected 48 h after transfection for further experiments. According to the manufacturer's instructions, OSCC cells were treated with Annexin V‐APC/PI Apoptosis (E‐CK‐A217; Elabscience, Wuhan, China) or Annexin V‐PE/7ADD Apoptosis (E‐CK‐A216; Elabscience, Wuhan, China) at room temperature in the dark, and then analysed by flow cytometry.

### 2.6 Public databases

The expression of CKIP‐1, the expression of TFAM in HNSCC were identified in the TCGA (https://cistrome.shiny apps.io/timer/; https://portal.gdc.cancer.gov/) database. The correlation between CKIP‐1 and EMT, and the correlation between CKIP‐1 and angiogenesis in HNSCC were identified in the assistant for clinical bioinformatics (ACLBI) database (https://www.aclbi.com/static/index.html#/). The expression of CKIP‐1 and TFAM in OSCC was also identified in the gene expression omnibus (GEO; GSE30784) database.

### 2.7 Cell transfection

OSCC cells were transfected with 50 nM small interfering RNAs (siRNAs; GenePharma, Suzhou, China) for CKIP‐1 transiently by GP‐transfect‐Mate (GenePharma, Suzhou, China) (Table S1). Lentiviruses containing a CKIP‐1‐specific short hairpin RNA (sh‐CKIP‐1; sense, 5’‐GGACCTGGTAGCAAGGAAA‐3′) as well as its control (sh‐NC; sense, 5’‐TTCTCCGAACGTGTCACGT‐3′) were also constructed by GenePharma (Suzhou, China) to obtain stable CKIP‐1‐silenced OSCC cell lines in the presence of 5 ng/mL polybrene (GenePharma, Suzhou, China). The transfected cells were cultured with 2 μg/mL puromycin for 7 days. Transient transfection of CKIP‐1‐overexpressing (CKIP‐1‐over) and control (NC‐over) plasmids (Miaoling, Wuhan, China) into OSCC cells was performed using TurboFect (Thermo Scientific, MA, USA) according to the manufacturer's instructions. The medium was changed after 8 h of transfection.

### 2.8 Quantitative real‐time polymerase chain reaction (RT‐qPCR)

Total RNA of SCC25 cells, CAL27 cells were extracted by a TRIzol extraction kit (Takara, Osaka, Japan) according to the manufacturer's protocol. The total RNA was reverse‐transcribed by the Takara RT‐PCR system (Takara, Osaka, Japan). Applied Biosystems QuantStudio 6 was used to perform qPCR with 10 μM specific primers in a 20 μL total reaction volume using SYBR qPCR Master Mix (Vazyme Biotech, Nanjing, China). These thermocycling conditions were implemented: 95°C for 30 s; 40 cycles at 95°C for 10 s and 62°C for 34 s; 72°C for 30 s. All signals were standardized to glyceraldehyde‐3‐phosphate dehydrogenase (GAPDH) and quantified using the 2^−ΔΔCt^ method. Specific primers (Sangon, Shanghai, China) were shown in Table S2.

### 2.9 Western blotting

Proteins were extracted from SCC25 and CAL27 cells by M‐PER mammalian protein extraction reagent (Thermo Scientific, MA, USA). SDS‐PAGE was used to separate protein lysates before they were transferred to polyvinylidene difluoride (PVDF) membranes (Millipore, NJ, USA). After blocking with 5% skimmed milk for 1 h at room temperature, the membranes were exposed to primary antibodies against Bax, Bcl2, CKIP‐1, Caspase 3, cGAS, CD9, CD81, E‐cadherin, GM130, Vimentin, TFAM, STING, TSG101, β‐actin at 4°C overnight. Next day, they were exposed to horseradish peroxidase (HRP)‐conjugated goat anti‐rabbit (1:8000; Proteintech, CA, USA) or goat anti‐mouse secondary antibodies (1:1000; Proteintech, CA, USA) at room temperature for 1 h, and visualized by the Odyssey LI‐COR scanner. Antibodies were shown in Table S3.

### 2.10 Ethynyl‐2‐deoxyuridine (EdU) assay

EdU detection was performed using the BeyoClick™ EdU Cell Proliferation Kit with Alexa Fluor 594 (Beyotime, Nanjing, China). 1 × 10^4^ OSCC cells were evenly seeded into each well of the 24‐well plates and cultured for 24 h. Cells were cultivated with the EdU reagents for 2 h, and then fixed with 4% paraformaldehyde (Servicebio, Wuhan, China). Cell staining was performed following the manufacturer's instruction. Images were obtained with a fluorescence microscope.

### 2.11 Cell counting kit‐8 (CCK‐8) assay

Each well in the 96‐well plates was inoculated with 2 × 10^3^ cells. After incubating with 100 μL complete medium plus 10 μL CCK‐8 reagent (Dojindo, Kyushu island, Japan) for 1 h at 37°C, the absorbance at 450 nm was measured using a microtiter plate. Each sample was evaluated in triplicate.

### 2.12 Colony formation assay

Two thousand cells were inoculated in the 6‐well plates and cultured in 10% FBS complete medium. Ten days later, colonies were stained with crystal violet (Solarbio, Beijing, China) for 30 min and washed three times with PBS. Finally, colonies larger than 1 mm in diameter were counted, and quantified.

### 2.13 Wound healing assay

1.2 × 10^5^ cells were seeded in the 24‐well plates. By the confluence stage, the cells were scratched with a 200 μL pipette tip to make uniform lengths, and then cultured in basal medium. The cells were washed with PBS to remove isolated cell debris, each wound was imaged at 0, 24, 36 h with a microscope. Cell migration was quantified using ImageJ.

### 2.14 Migration and invasion assay

OSCC cells were inoculated in the upper chamber with (invasion assay; Corning, NY, USA) or without (migration assay; Corning, NY, USA) Matrigel (BD, NJ, USA), and were induced to migrate or invaded to the lower chamber for 24 h. After wiping the upper layer of transwell membrane, the migrated cells were stained with crystal violet (Solarbio, Beijing, China) for 20 min, and observed with a microscope.

### 2.15 Immunohistochemistry (IHC)

Tissues were fixed with 4% paraformaldehyde, paraffin‐embedded, and then sectioned. HE staining was applied to identify the OSCC tissues. PBS was used as the negative control for IHC. Primary antibodies against CKIP‐1 was shown in Table S3. After antigen repairing, the sections were stained for IHC by incubating with the reagents of the UltraSensitive SP IHC Kit (MXB Biotech, Fuzhou, China) following the manufacturer's protocol. All slides were scanned by CaseViewer 2.4 (3DHISTECH Ltd., Budapest, Hungary).

### 2.16 Immunofluorescence (IF)

Cells were fixed with 4% paraformaldehyde for 15 min, and permeated with 0.2% Triton X‐100 (Beyotime, Nanjing, China) for 5 min. Primary antibodies against E‐cadherin, Vimentin, Ki67, TFAM and CKIP‐1 were shown in Table S3. After incubated with the primary antibodies overnight, the cell or tissue samples were incubated with fluorescent secondary antibodies for 1 h, and then DAPI (Beyotime, Nanjing, China) for 5 min. The sealed samples were observed and photographed with a microscope.

### 2.17 Mitochondrial ROS measurement

MitoSOX™ Red mitochondrial superoxide indicator (YEASEN, Shanghai, China) was used to measure mitochondrial ROS according to the instructions from the manufacturer. Briefly, 1.5 × 10^4^ OSCC cells were seeded in the 48‐well plates. After treated with 10 μM TMP (Sigma‐Aldrich, MO, USA) for 72 h, OSCC cells were incubated with 2.5 μM MitoSOX™ reagent working solution for 20 min in the dark. After washed with HBSS, fixed with 4% paraformaldehyde and stained with DAPI (Beyotime, Nanjing, China), ROS was detected by a fluorescent microscope.

### 2.18 Mitochondrial membrane potential detection

MitoTracker™ Red CMXRos (Invitrogen, CA, USA) was used to measure the mitochondrial membrane potential according to the instructions of the manufacturer. Briefly, 1.5 × 10^4^ OSCC cells were seeded in the 48‐well plates. After treated with 10 μM TMP (Sigma‐Aldrich, MO, USA) for 72 h, OSCC cells were incubated with 200 nM MitoTracker™ reagent working solution for 30 minutes in the dark. After washed with HBSS, fixed with 4% paraformaldehyde, stained with Acti‐stain™ 488 phalloidin (DENVER, CO, USA) and DAPI (Beyotime, Nanjing, China), mitochondrial membrane potential was detected by a fluorescent microscope.

### 2.19 Cell‐derived xenograft (CDX) tumour model

This study conformed to the updated ARRIVE 2.0 guidelines for preclinical animal studies. To investigate the role of CKIP‐1 in OSCC in vivo, we established xenograft tumours in 5‐week‐old female BALB/c nude mice purchased from the Model Animal Research Center of Wuhan University. CKIP‐1 knockdown CAL27 cells (2 × 10^6^) and their control cells were implanted subcutaneously in nude mice (*n* = 5/group). The length (*L*) and width (*W*) of the tumours were measured with a Vernier calliper, and the tumour volume (*V*) was calculated according to the formula *V* = *L* × *W* ^2^ × 0.5. Tumour size was measured every 7 days after cell implantation. The mice were killed on Day 28 by the overdose of anaesthesia. Tumours were photographed and weighed, and then fixed in 4% paraformaldehyde for further experiments. Hepatic metastasis was also photographed and quantified. All experimental procedures were approved by the Animal Protection and Use Committee of Wuhan University.

### 2.20 mRNA sequencing

mRNA samples of CKIP‐1‐silenced CAL27 cells and their control CAL27 cells in three biological repeats were extracted using TRIzol reagent (Takara, Osaka, Japan). After quality control and purification, the samples were sent to Beijing Genomic Institute (BGI, Beijing, China) for library construction and sequencing. The expression level of gene was calculated by RPKM (Reads per Kilobase per Million Reads). The GO and KEGG enrichment analysis of annotated different expressed gene was performed on the platform (https://mybgi.bgi.com/).

### 2.21 Chemical drugs

In this study, 2, 3, 5, 6‐tetramethylpyrazine (TMP; Sigma‐Aldrich, MO, USA) was used as a drug to inhibit the degradation of TFAM at a concentration of 10 μM for 24 h.

### 2.22 Statistical analysis

Statistical analysis was performed with GraphPad Prism 8 software (CA, USA). Comparisons between groups were analysed using the unpaired Student's *t*‐test and one‐way ANOVA tests followed by Dunnett's multiple comparison tests. Association between CKIP‐1 and TFAM expression was calculated by the Pearson or Spearman analysis. Results were presented as means ± SD. Significance was defined as *p < 0.05, **p < 0.01, ***p < 0.001 and ****p < 0.0001.

## 3 RESULTS

### 3.1 CKIP‐1 was upregulated in OSCC tissues and cell lines

**FIGURE 1:**
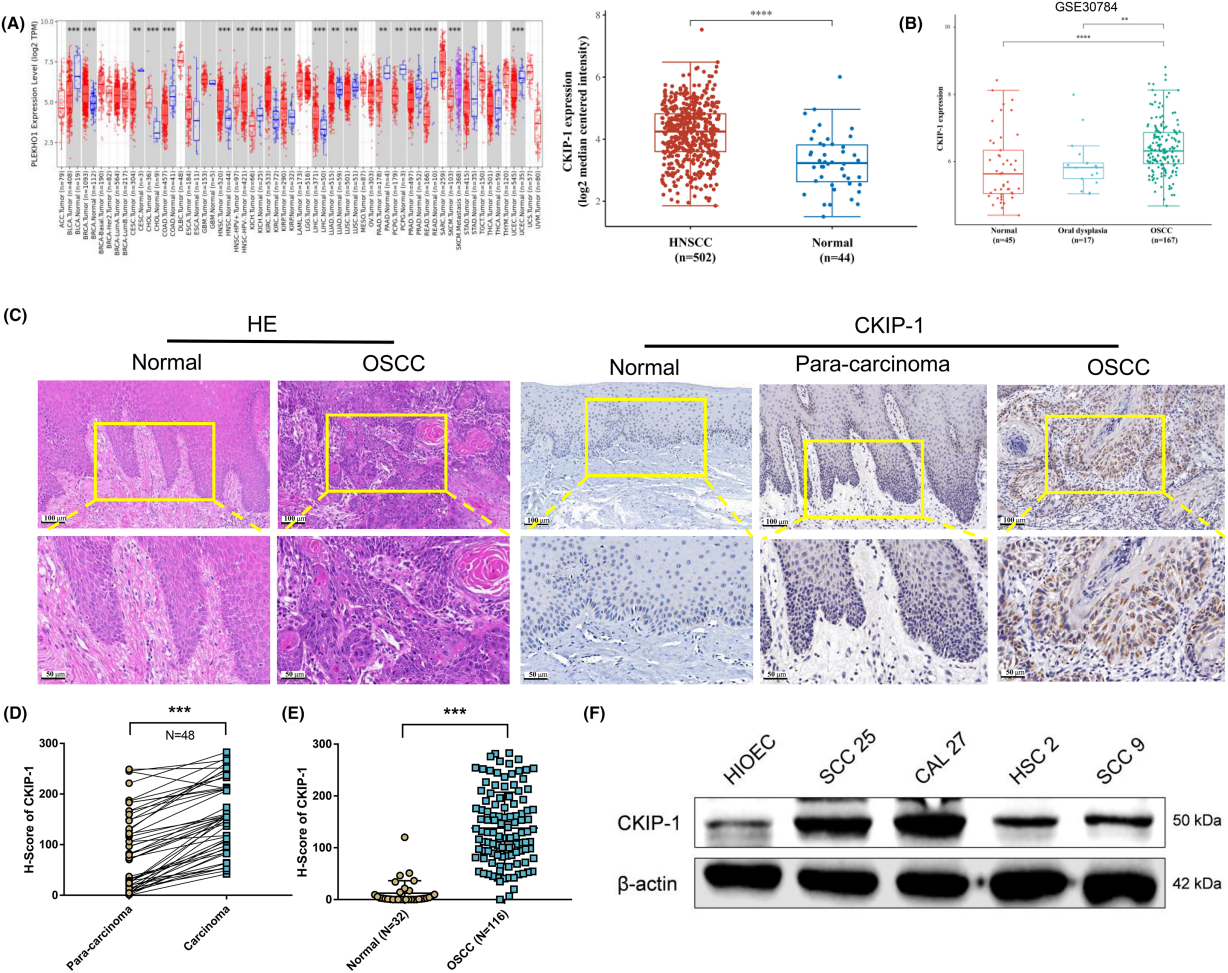
CKIP‐1 was upregulated in OSCC tissues and cell lines. Higher expression of CKIP‐1 in HNSCC and OSCC compared with normal tissues was observed in the TCGA (A) and GEO (B) databases, respectively. OSCC tissues were identified by HE staining. Higher expression of CKIP‐1 in OSCC tissues compared with normal tissues and paired adjacent tissues was demonstrated by immunohistochemistry. Representative images (C), and quantitative analysis (D, E)002E Higher expression of CKIP‐1 in SCC25, CAL27, HSC2 and SCC9 cell lines compared with normal HIOECs was found by Western blotting (F). Scale bar: 100 μm for the upper pictures of (C), and 50 μm for the lower pictures of (C). Significance was defined as **p < 0.01, ***p < 0.001 and ****p < 0.0001.

First, higher expression of CKIP‐1 in HNSCC and OSCC tissues compared with the normal tissues was found in the TCGA database (Figure 1A) and the GEO database (Figure 1B), respectively. Then, 116 OSCC tissue samples (48 samples have paired paraneoplastic tissues) and 32 normal tissue samples were collected for immunohistochemistry of CKIP‐1. Light staining of CKIP‐1 in normal tissues, moderate staining of CKIP‐1 in OSCC paraneoplastic tissues, and heavy staining of CKIP‐1 in OSCC tissues were detected (Figure 1C). The negative control for IHC of CKIP‐1 in normal and OSCC tissues was also performed (Figure S1). Quantification results also showed higher CKIP‐1 expression in OSCC tissues when compared with the tumour‐adjacent tissues (Figure 1D) or the normal tissues (Figure 1E). Meanwhile, the protein expression of CKIP‐1 in 4 OSCC cell lines (SCC9, SCC25, CAL27, and HSC2) and HOIEC was examined. Higher CKIP‐1 expression in all OSCC cell lines was found when compared with HOIEC, with the highest level in SCC25 and CAL27 cells (Figure 1F). Therefore, SCC25 and CAL27 cells were selected for the subsequent experiments. Taken together, CKIP‐1 was found to be increased in OSCC tissues and cell lines.

### 3.2 CKIP‐1 positively regulated the proliferation, migration, invasion and negatively regulated the apoptosis of OSCC cells

**FIGURE 2:**
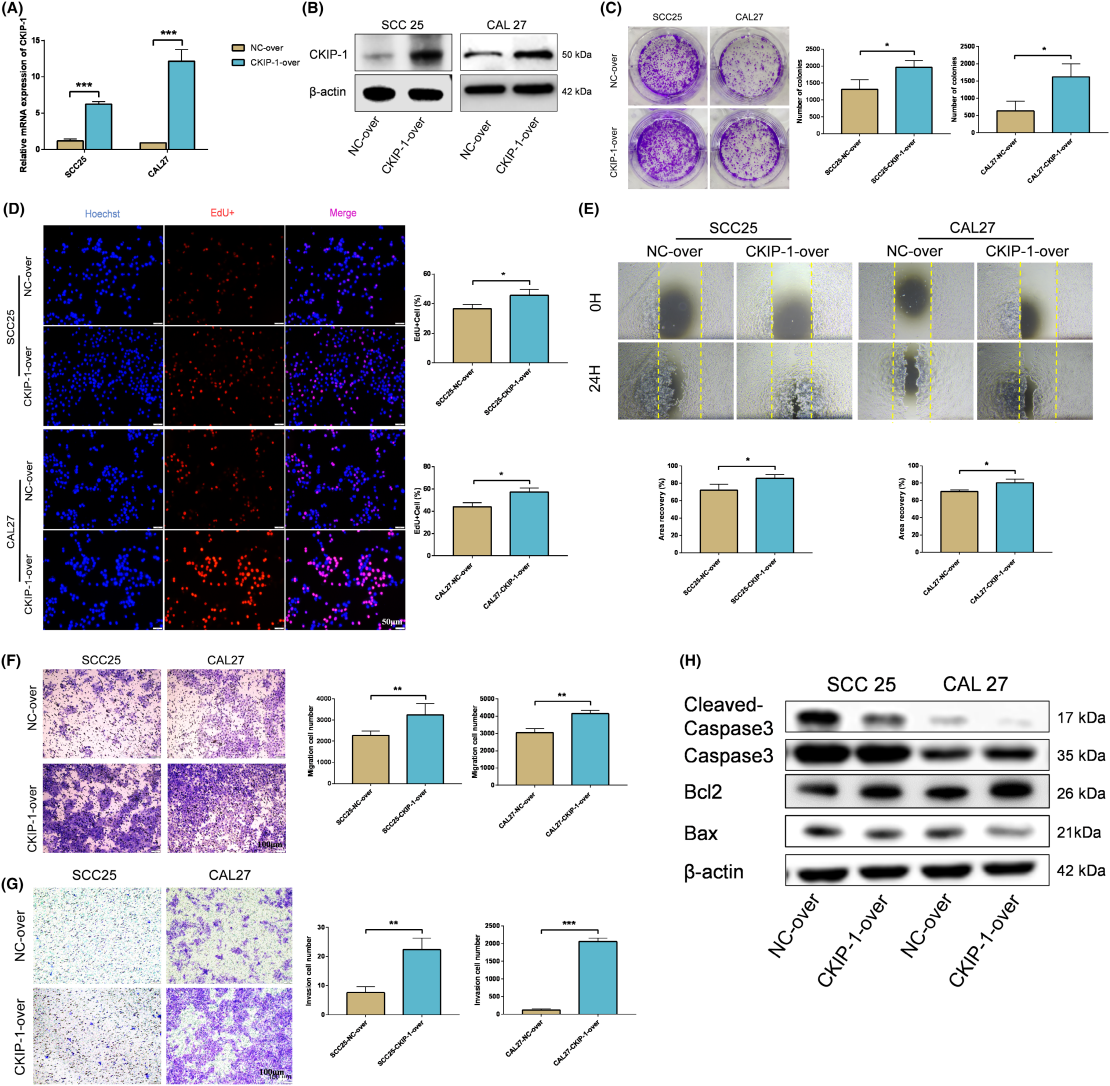
CKIP‐1 overexpression promoted proliferation, migration, invasion and inhibited the apoptosis of OSCC cells. The transient overexpression efficiency of CKIP‐1 in OSCC cells was verified by RT‐qPCR (A) and Western blotting (B) Enhanced proliferation of CKIP‐1‐overexpressed OSCC cells compared with control cells were detected by colony formation assay (C), EdU assay (D). Increased migration of CKIP‐1‐overexpressed OSCC cells compared with control cells were observed by wound healing assay (E) and transwell migration assay (F). Enhanced invasion of CKIP‐1‐overexpressed OSCC cells compared with control cells were examined by Matrigel invasion assay (G). Downregulation of cleaved‐Caspase 3, Bax, upregulation of Bcl2 in CKIP‐1‐overexpressed OSCC cells compared with control cells were detected by Western blotting (H). Magnification: 4× for (E). Scale bar: 100 μm for (F, G); 50 μm for (D). Significance was defined as *p < 0.05, **p < 0.01 and ***p < 0.001.

**FIGURE 3:**
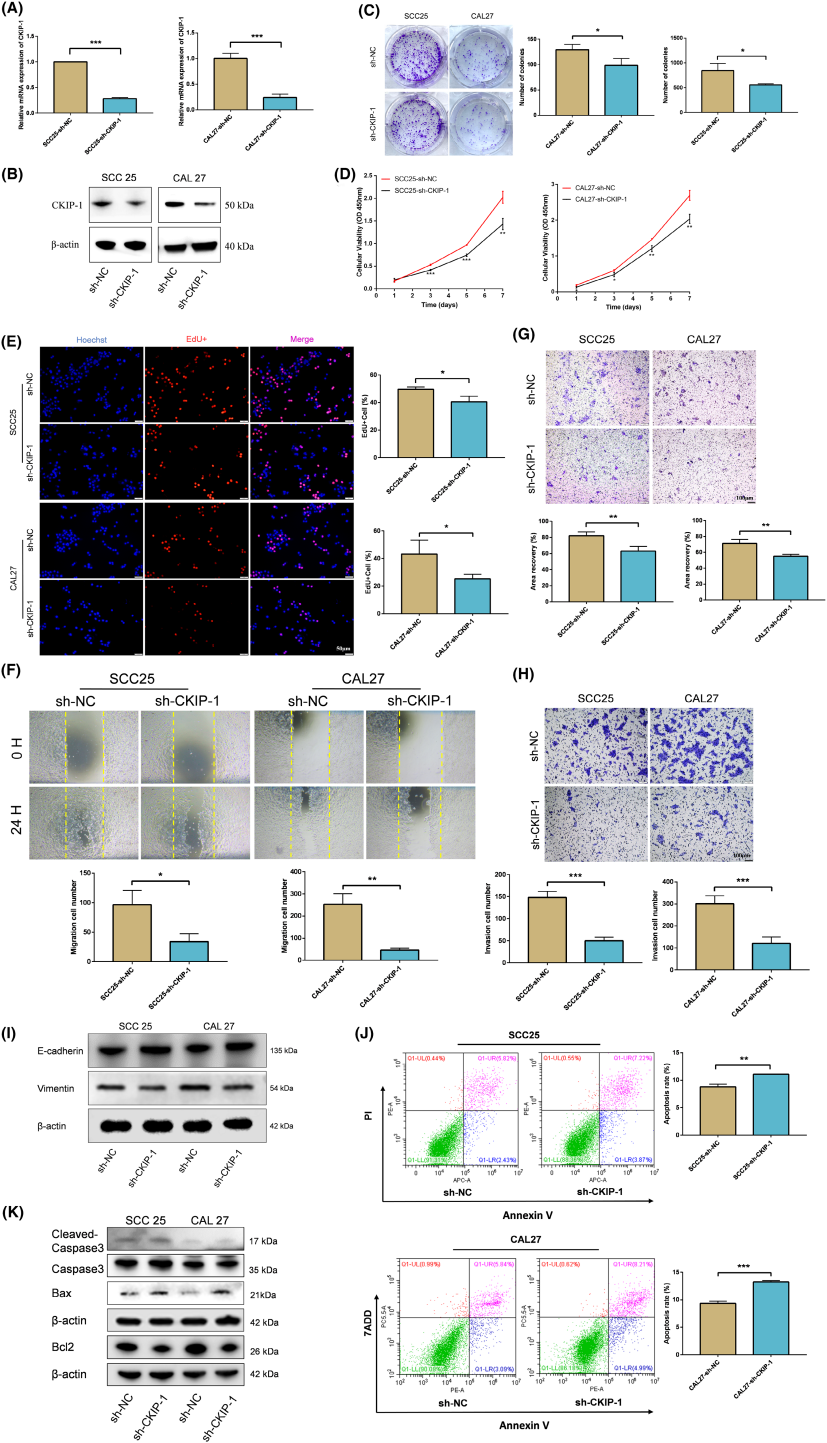
CKIP‐1 silencing inhibited proliferation, migration, invasion and promoted the apoptosis of OSCC cells. The knockdown efficiency of CKIP‐1 in OSCC cells was verified by RT‐qPCR (A) and Western blotting (B). Inhibited proliferation of CKIP‐1‐silenced OSCC cells compared with control cells were detected by colony formation assay (C), CCK‐8 assay (D) and EdU assay (E). Decreased migration of CKIP‐1‐silenced OSCC cells compared with control cells were observed by wound healing assay (F) and transwell migration assay (G). Inhibited invasion of CKIP‐1‐silenced OSCC cells compared with control cells were examined by Matrigel invasion assay (H). Upregulation of E‐cadherin, downregulation of Vimentin in CKIP‐1‐silenced OSCC cells compared with the control cells were detected by Western blotting (I). Increased apoptosis in CKIP‐1‐silenced OSCC cells compared with the control cells was verified by flow cytometry (J). Upregulation of cleaved‐Caspase 3, Bax, downregulation of Bcl2 in CKIP‐1‐silenced OSCC cells compared with control cells were detected by Western blotting (K). Magnification: 4× for (F). Scale bar: 100 μm for (G, H); 50 μm for (E). Significance was defined as *p < 0.05, **p < 0.01 and ***p < 0.001.

CKIP‐1‐overexpressed OSCC cells were constructed by transfecting with plasmids overexpressing CKIP‐1, and identified successfully (Figure 2A,B). CKIP‐1‐silenced OSCC cells were constructed by transient siRNA (si‐CKIP‐1‐homo‐1434 with the best knockdown efficiency was finally selected) transfection (Figure S2A,B) and stable lentivirus transfection (Figure S3; Figure 3A,B), and identified successfully. Increased proliferation of CKIP‐1‐overexpressed OSCC cells (Figure 2C,D) and decreased proliferation of CKIP‐1‐silenced OSCC cells (Figure S2C,D; Figure 3C–E) were detected by the plate cloning, EdU and CCK8 assays. Also, wound healing, transwell‐migration and Matrigel invasion assays showed that the migration and invasion capacity of OSCC cells increased after CKIP‐1 overexpression (Figure 2E–G), whereas decreased after CKIP‐1 silencing (Figure S2E,F,G; Figure 3F–H). In addition, upregulated expression of E‐cadherin, downregulated expression of Vimentin in CKIP‐1‐silenced OSCC cells were examined by Western blotting (Figure 3I) and immunofluorescence (Figure S4A,B). The public database also showed a positive correlation between CKIP‐1 expression and epithelial‐mesenchymal transition (EMT) in HNSCC (Figure S4C). Moreover, increased apoptosis in CKIP‐1‐silenced OSCC cells was determined by flow cytometry (Figure 3J), PI staining (Figure S5A) and TUNEL staining (Figure S5B). Western blotting also showed lower expression of cleaved‐Caspase 3, Bax, and higher expression of Bcl2 in CKIP‐1‐overexpressed group when compared with the control group (Figure 2H), and contrary results were found in CKIP‐1‐silenced group (Figure S2H; Figure 3K). In all, CKIP‐1 positively regulated the malignant behaviours of OSCC cells.

### 3.3 CKIP‐1 silencing inhibited tumour growth in vivo

**FIGURE 4:**
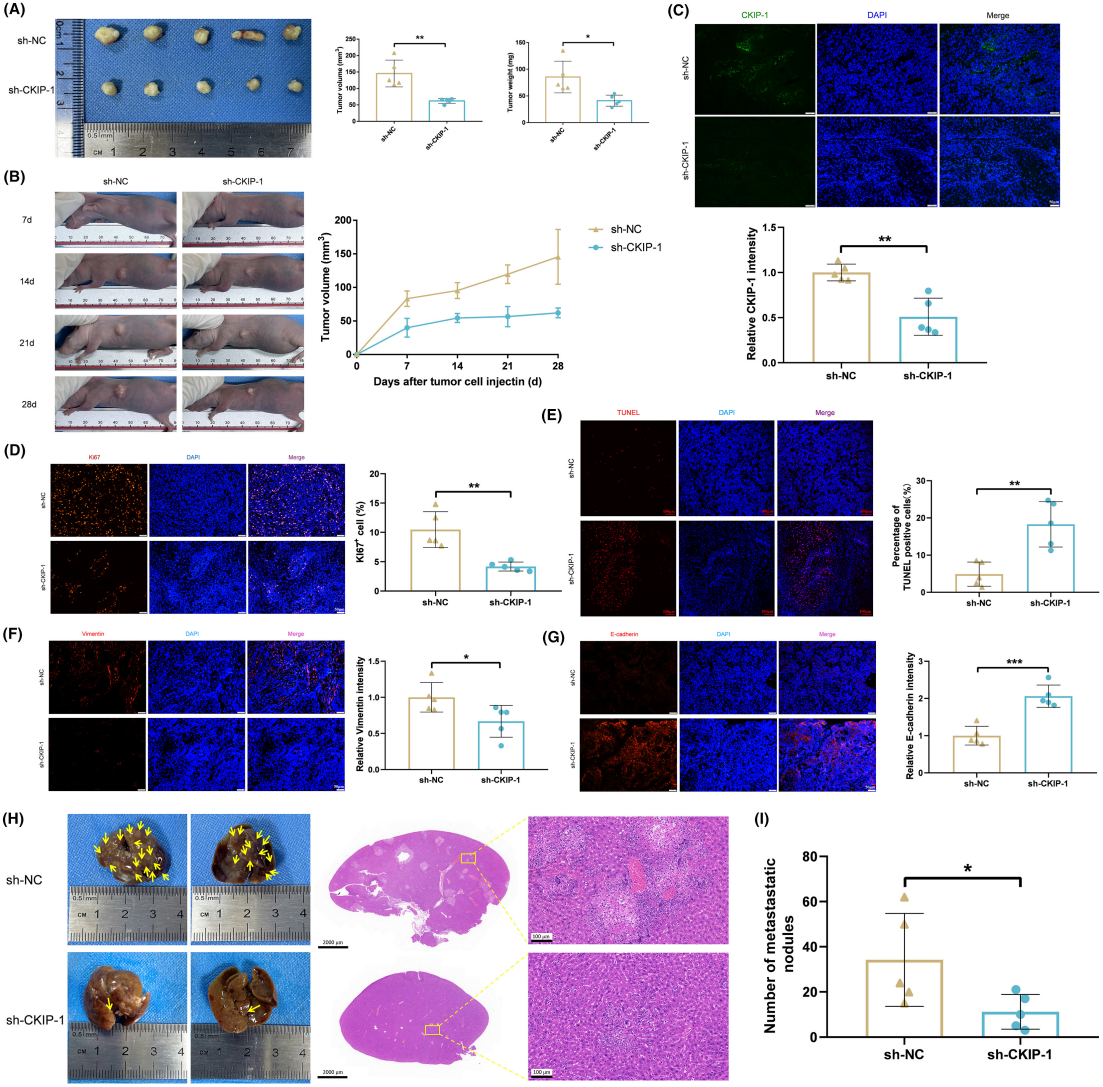
CKIP‐1 silencing inhibited tumour growth in vivo. The weight and volume of tumours in sh‐CKIP‐1 group were significantly less than those in sh‐NC group (A). The growth curve of the in vivo tumorigenicity assay showed that the subcutaneous tumour growth in sh‐CKIP‐1 group was significantly slower (B). Immunofluorescence of mice tumours showed lower expression of CKIP‐1 in sh‐CKIP‐1 group compared with that in sh‐NC group (C). Immunofluorescence of mice tumours showed lower expression of Ki67 (D), vimentin (F), and higher expression of E‐cadherin (G) in sh‐CKIP‐1 group compared with that in sh‐NC group. TUNEL staining of mice tumours showed less TUNEL positive cells in sh‐CKIP‐1 group than in sh‐NC group (E). Higher liver metastases were found in sh‐CKIP‐1 group than that in sh‐NC group by gross observation and HE staining (H, I). Scale bar: 50 μm for (C, D, F, G); 100 μm for (E); 2000 and 100 μm for (H). Significance was defined as *p < 0.05, **p < 0.01 and ***p < 0.001.

The antitumor activity of CKIP‐1 silencing in oral squamous cell carcinogenesis was examined by a nude mouse subcutaneous xenograft model, in which sh‐NC and sh‐CKIP‐1 CAL27 cells was applied. We observed the status of nude mice and recorded the tumour size every 7 days, and dissected the tissues on Day 28. The tumour volume and weight of mice was significantly reduced in sh‐CKIP‐1 group compared to the control group on day 28 (Figure 4A). Also, smaller tumour size in sh‐CKIP‐1 group was found at every recording time point (Figure 4B). By means of immunofluorescence, lower expression of CKIP‐1 in sh‐CKIP‐1 group compared with control group was confirmed (Figure 4C). Besides, decreased proliferation and increased apoptosis in sh‐CKIP‐1 group were verified by Ki67 staining and TUNEL staining, respectively (Figure 4D,E). For EMT markers, elevated E‐cadherin expression and decreased Vimentin expression in sh‐CKIP‐1 group was also demonstrated (Figure 4F,G). These results suggested that CKIP‐1 silencing reduced the tumorigenic capacity of CAL27 in vivo. Moreover, multiple metastases in the liver of nude mice were observed in sh‐NC group. However, the liver metastasis sites significantly reduced after CKIP‐1 silencing (Figure 4H,I). To sum up, both in vitro and in vivo experiments indicated that CKIP‐1 silencing suppressed OSCC progression.

### 3.4 CKIP‐1 silencing inhibited OSCC progression through TFAM/cGAS‐STING signalling axis

**FIGURE 5:**
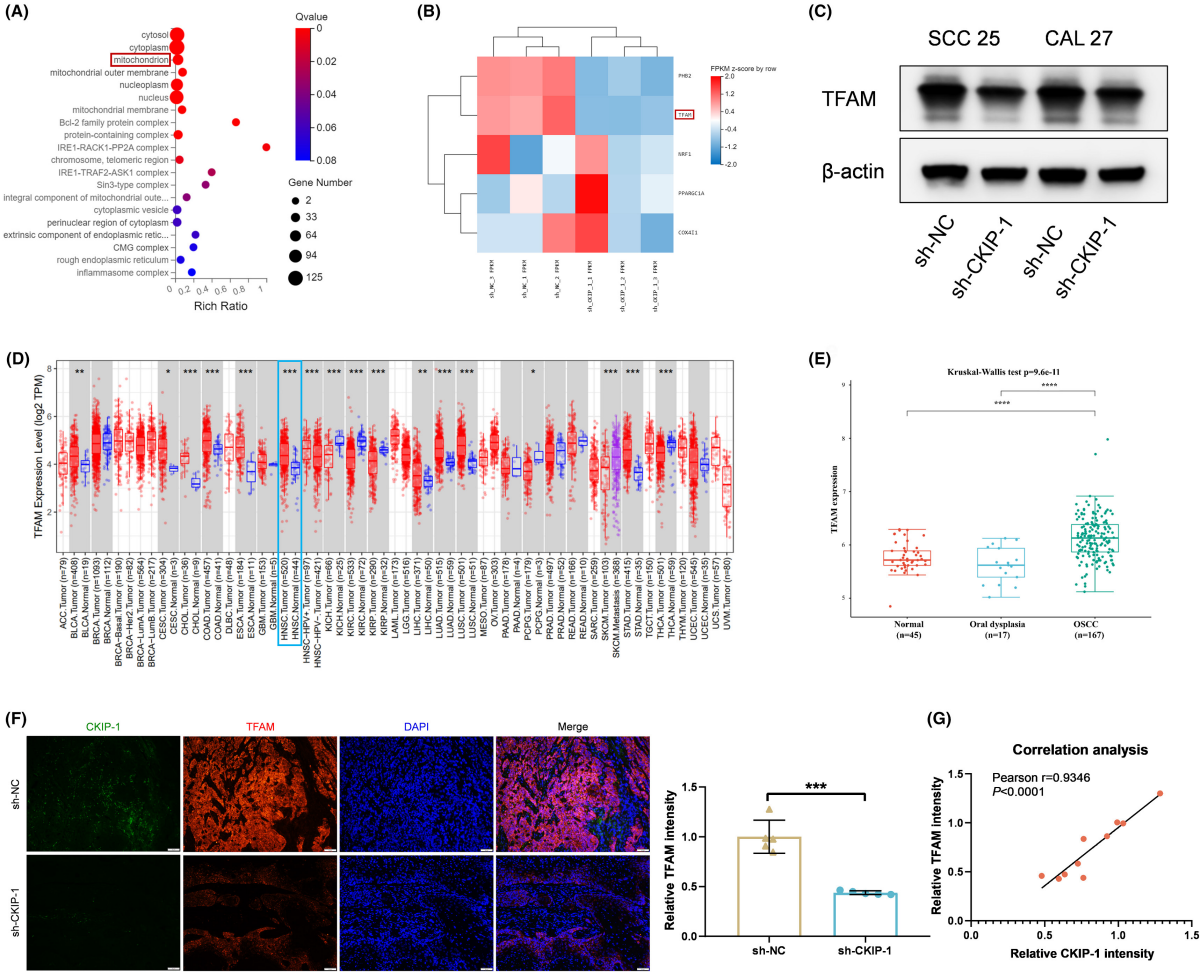
CKIP‐1 silencing inhibited TFAM expression in OSCC cells. GO analysis showed that differentially expressed genes could be enriched in mitochondria (A). Heat map analysis of mitochondrial biogenesis‐related genes showed significant downregulation of TFAM in OSCC cells when CKIP‐1 was silenced (B). Downregulation of TFAM in CKIP‐1‐silenced OSCC cells were verified by Western blotting (C). Higher TFAM expression was found in HNSCC and OSCC tumour tissues than that in normal tissues in TCGA and GEO (GSE30784) databases, respectively (D, E). Double immunofluorescence of CKIP‐1 and TFAM in mice tumours showed that the expression of TFAM was significantly decreased in sh‐CKIP‐1 group (F), and was positively correlated with the expression of CKIP‐1 (G). Scale bar: 50 μm for (F). Significance was defined as *p < 0.05, **p < 0.01, ***p < 0.001 and ****p < 0.0001.

In order to explore the underlying mechanism in CKIP‐1 silencing‐suppressed OSCC progression, mRNA sequencing was applied. GO analysis indicated that the differentially expressed genes could be enriched in mitochondria (Figure 5A). Among the mitochondrial biogenesis‐related genes, heatmap analysis further revealed a significant reduction expression of TFAM in sh‐CKIP‐1 group, an essential gene involved in mitochondria (Figure 5B). The downregulation of TFAM in CKIP‐1‐silenced OSCC cells was also confirmed by Western blotting (Figure 5C).

**FIGURE 6:**
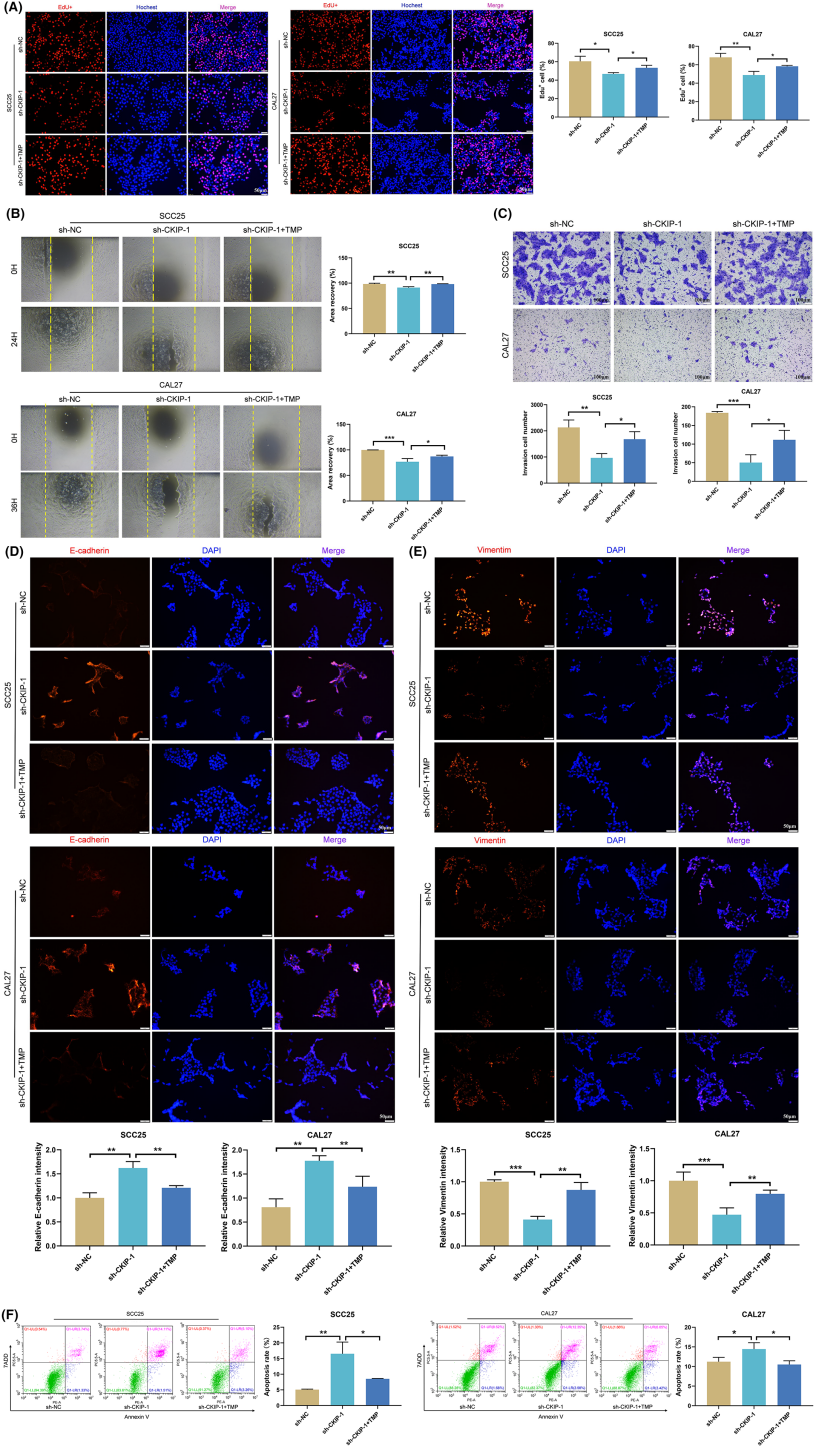
Inhibition of TFAM degradation reversed the anti‐tumour effect induced by CKIP‐1 silencing. Suppressed proliferation (A), migration (B), invasion (C), Vimentin expression (E) and increased E‐cadherin expression (D), apoptosis (F) in sh‐CKIP‐1 group were reversed by TMP, a drug that inhibits TFAM degradation. Magnification: 4× for (B). Scale bar: 100 μm for (C); 50 μm for (A, D, E). Significance was defined as *p < 0.05, **p < 0.01 and ***p < 0.001.

Then, increased expression of TFAM in HNSCC and OSCC compared with the normal tissues in the TCGA and the GEO databases was reviewed, respectively (Figure 5D,E). Consistent with this, TFAM expression was also significantly reduced in the nude mice tumour samples in sh‐CKIP‐1 group (Figure 5F). Also, TFAM expression was demonstrated to be positively correlated with the expression of CKIP‐1 (Figure 5G). Therefore, the regulating role of TFAM in CKIP‐1‐silenced OSCC cells was then studied. The effects of TMP, a drug well‐documented to have a role in inhibiting TFAM degradation,^19^ was first verified in CKIP‐1‐silenced OSCC cells by Western blotting (Figure S6). After TMP addition, CKIP‐1 silencing‐suppressed proliferation (Figure 6A), migration (Figure 6B) and invasion (Figure 6C), and CKIP‐1 silencing‐enhanced apoptosis (Figure 6F) of OSCC cells were all reversed, as well as the expression of E‐cadherin (Figure 6D) and Vimentin (Figure 6E). These results suggested that CKIP‐1 silencing‐induced anti‐tumour effects could be exacerbated by TFAM, a potential positive regulator of OSCC.

**FIGURE 7:**
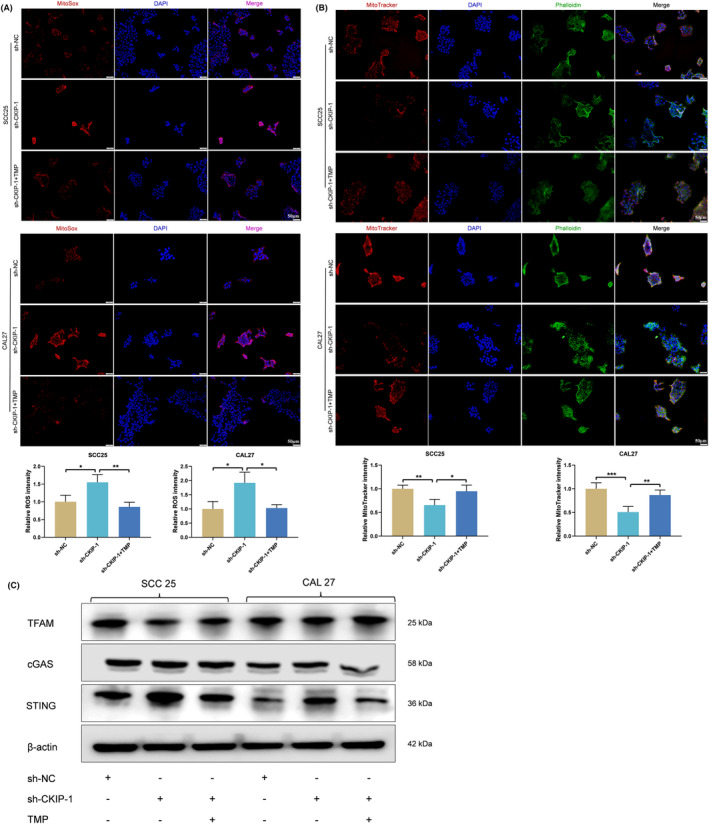
Inhibition of TFAM degradation reversed CKIP‐1 silencing‐induced mitochondrial dysfunction and cGAS‐STING activation. Suppressed mitochondrial membrane potential (A) and increased ROS production (B) in sh‐CKIP‐1 group were reversed by TMP, a drug that inhibits TFAM degradation. Activation of cGAS‐STING signalling pathway in OSCC cells was weakened by TMP (C). 50 μm for (A, B). Significance was defined as *p < 0.05, **p < 0.01 and ***p < 0.001.

Furthermore, increased ROS production and decreased mitochondrial membrane potential in CKIP‐1‐silenced OSCC cells was also reversed after TMP was added (Figure 7A,B), indicating a weaker anti‐tumour effect. Due to the reason that TFAM degradation could activate the cGAS‐STING axis to inhibit OSCC, the downstream cGAS‐STING pathway was further evaluated by Western blotting. The results showed that TFAM degradation triggered by CKIP‐1 silencing was positively correlated with the activation of cGAS‐STING axis, while this activation was inhibited after the addition of TMP (Figure 7C). Taken together, these results demonstrated that CKIP‐1 silencing could function anti‐tumour effect on OSCC cells partially via TFAM/cGAS‐STING signalling axis.

## 4 DISCUSSION

In this study, CKIP‐1 was demonstrated to be an indicator positively correlated with OSCC. In vitro gain‐ and loss‐of‐function experiments and in vivo CDX model were applied. From a therapeutic point of view, CKIP‐1 silencing could suppress the proliferation, migration, invasion and tumour growth, and promoted the apoptosis in OSCC cells and transplanted tumour tissues. Moreover, the mechanisms underlying CKIP‐1 silencing could inhibit the malignant behaviours of OSCC cells directly via TFAM/cGAS‐STING axis. In all, our study provides new insights into the role and mechanism of CKIP‐1 in OSCC.

CKIP‐1 possesses different domains which facilitates the interaction with many signals to be involved in various diseases, such as osteoporosis, cardiac hypertrophy, immune disorders, atherosclerosis and tumour.^7^, ^8^, ^20^ In initial studies, CKIP‐1 seems to be well documented to be a tumour suppressor. Zhu et al. found that CKIP‐1 silencing promoted the proliferation of non‐Hodgkin's lymphoma cells by interacting with and restraining AKT signalling pathway.^10^ Nie et al. indicated that CKIP‐1 inhibited cell growth, migration and colonic tumour formation by suppressing the production and enhanced the auto‐degradation of oncogenic Smurf1.^11^ Ma et al. proved that CKIP‐1 overexpression downregulated the Ras/ERK pathway to promote apoptosis in the intestinal type of gastric cancer.^13^ Xi et al. demonstrated that CKIP‐1 could suppress AKT/GSK3β/β‐catenin signalling pathway to function the anti‐proliferative and pro‐apoptotic effects in glioma cells.^14^ However, some studies have shown that CKIP‐1 could play a pro‐oncogenic role in certain tumours. Yu et al. found that CKIP‐1 knockdown inhibited the activity of renal cell carcinoma cells in vitro and in vivo via Hippo and MAPK/JNK pathways.^21^ Chen et al. confirmed that CKIP1 gene promoted proliferation and inhibited apoptosis of human lung cancer cell line H1299.^22^ These studies suggested that CKIP‐1 plays different roles in different tumour types. In our study, we found that CKIP‐1 expression was upregulated in patient OSCC tissues and cell lines (especially SCC25 and CAL27) and the tumour‐promoting role of CKIP‐1 in OSCC progression was also demonstrated. We speculated that this inconformity was mainly due to the different tumour model types, as this phenomenon has also been found in other genes. For example, Wilms' tumour 1 (WT1) gene was initially discovered to behave as a tumour suppressor in kidney tumours. However, WT1 gene was highly expressed in leukaemia, BRCA and many other tumours with an oncogenic role.^23^ Thus, it can be understood that one gene may exert different functions in different tumours. Consistently, from the results in TCGA database, increased CKIP‐1 expression was also found in BRCA, cholangiocarcinoma (CHOL), kidney renal clear/papillary cell carcinoma (KIRC/KIRP), liver hepatocellular carcinoma (LIHC), implying that CKIP‐1 may also be implicated in regulating the progression of these types of tumours positively.

TFAM is a mtDNA packaging protein to be the downstream signal in PGC‐1alpha/NRF/TFAM signalling pathway that required for mitochondrial biogenesis.^24^ In our OSCC models, CKIP‐1 silencing‐induced TFAM disruption disturbed the mitochondrial homeostasis characterized by decreased mitochondrial membrane potential and increased ROS production. The mitochondrial dysfunction was reserved when TMP, a drug that prevents TFAM degradation, was applied. Moreover, TFAM‐related mitochondrial dysfunction was confirmed to further activate the downstream cGAS‐STING axis, which was well‐documented to function a suppression effect on OSCC and many other tumours.^17^, ^18^, ^25^ Indeed, the activation of cGAS and STING was observed in CKIP‐1‐silenced OSCC cells. When adding TMP, the activation was weakened. These results are in keeping with other's work that targeting mitochondria to elicit its' dysfunction may be a potential therapeutic approach for OSCC treatment.^26^ Together, CKIP‐1 silencing‐suppressed OSCC progression was exacerbated after TMP addition, indicating the positive correlation between TFAM and OSCC development. In accordance with this, increased TFAM expression in HNSCC and OSCC was found in TCGA and GEO databases. Similar results were also demonstrated in hepatocellular carcinoma and breast cancer.^15^, ^16^ Therefore, we finally draw the conclusion that CKIP‐1 silencing could inhibit OSCC progression via TFAM/cGAS‐STING axis.

In our study, CKIP‐1 downregulation in OSCC cells activated the cGAS‐STING pathway. While this pathway is known to activate the NF‐kB signalling pathway and trigger the type‐I IFN signalling cascade,^27^ further investigation is needed to determine if the cGAS‐STING pathway activation induced by CKIP‐1 downregulation in OSCC cells results in elevated levels of NF‐κB and type‐I IFN. Future studies can involve analysing the inflammatory gene signature in these cells for a more comprehensive understanding.

## 5 CONCLUSIONS

In conclusion, our study demonstrated that CKIP‐1 silencing significantly inhibited OSCC progression via TFAM/cGAS‐STING signalling axis and elucidated the roles of CKIP‐1 in OSCC, which may provide a new target for OSCC treatment.

## AUTHOR CONTRIBUTIONS

**Ji‐Rong Xie:** Conceptualization (lead); data curation (lead); methodology (lead); project administration (lead); software (lead); visualization (lead); writing – original draft (lead); writing – review and editing (lead). **Xiao‐Jie Chen:** Software (supporting); visualization (supporting). **Gang Zhou:** Conceptualization (lead); data curation (supporting); methodology (supporting); project administration (lead); resources (lead); writing – review and editing (lead).

## FUNDING INFORMATION

This study was supported by grants from the National Natural Science Foundation of China to Gang Zhou (No. 82270983 and No. 81970949).

## CONFLICT OF INTEREST STATEMENT

The authors declare no competing interests.

## CONSENT FOR PUBLICATION

All authors consent to publication.

## Supporting information


Appendix S1:

## Data Availability

The data that support the findings of this study are available on request from the corresponding author. The data are not publicly available due to privacy or ethical restrictions.
