# Supplementary material for: CKIP‐1 silencing suppresses OSCC via mitochondrial homeostasis‐associated TFAM/cGAS‐STING signalling axis
Source: J Cell Mol Med. 2024 Aug 21;28(16):e70006. doi: 10.1111/jcmm.70006 (PMC11338841; doi:10.1111/jcmm.70006)
Supplement: Supplementary file 1 — Appendix S1: [file JCMM-28-e70006-s001.docx]

**CKIP-1 silencing suppresses OSCC via mitochondrial homeostasis-associated TFAM/cGAS-STING signaling axis**

Ji-Rong Xie, Xiao-Jie Chen, Gang Zhou


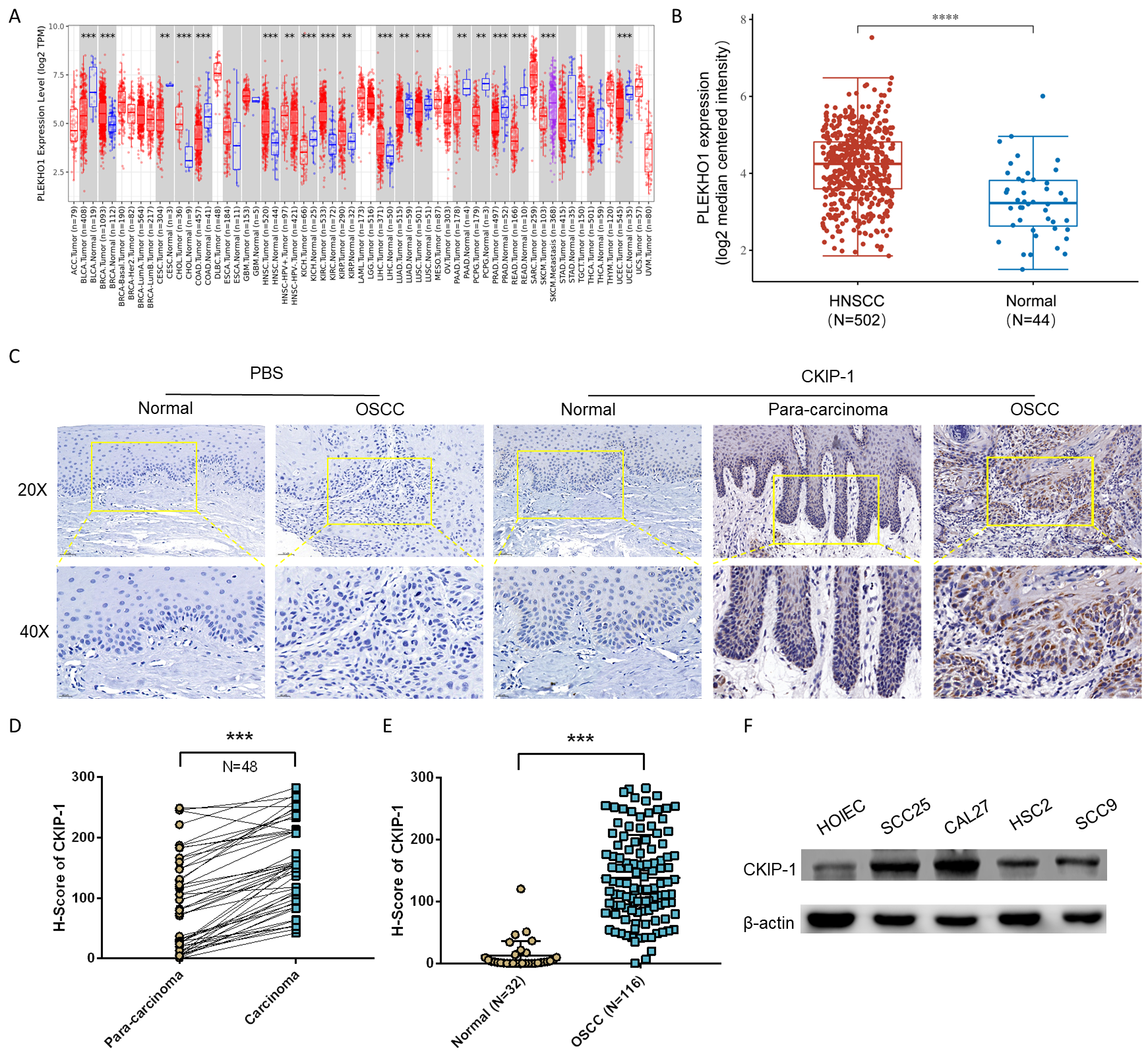


**Figure S1:** The negative control for IHC of CKIP-1 in normal and OSCC tissues.


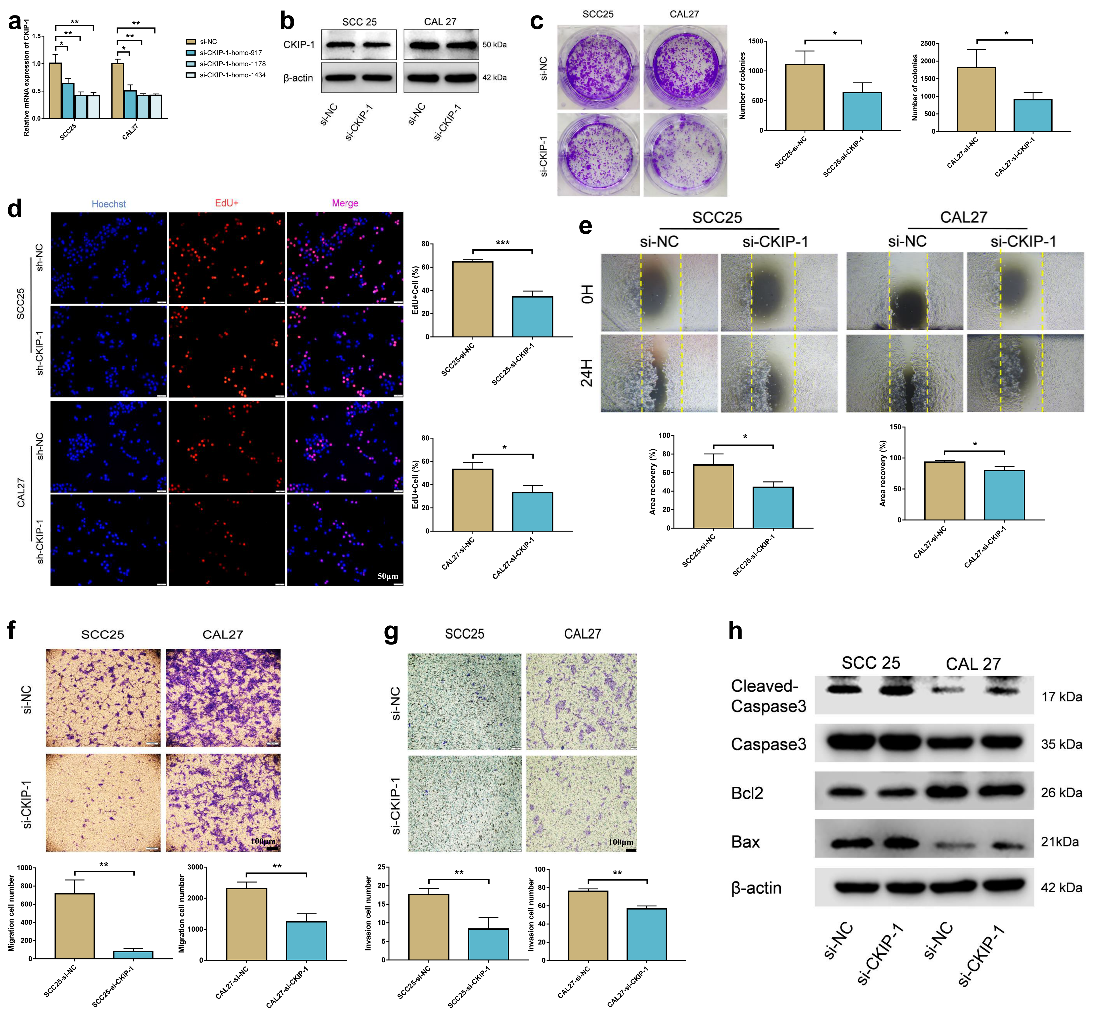


**Figure S2:** The transient knockdown efficiency of CKIP-1 in OSCC cells was verified by RT-qPCR **(a)** and Western blotting **(b)**. Suppressed proliferation of CKIP-1-silenced OSCC cells compared with control cells were detected by colony formation assay **(c)**, EdU assay (d). Decreased migration of CKIP-1-silenced OSCC cells compared with control cells were observed by wound healing assay **(e)** and transwell migration assay **(f)**. Inhibited invasion of CKIP-1-silenced OSCC cells compared control cells were examined by Matrigel invasion assay (g). Upregulation of cleaved-Caspase 3, Bax, downregulation of Bcl2 in CKIP-1-silenced OSCC cells compared with control cells were detected by Western blotting (h). Magnification: 4× for (e). Scale bar: 50 μm for (d); 100 μm for (f, g). Significance was defined as **P* < 0.05, ***P* < 0.01 and ****P* < 0.001.


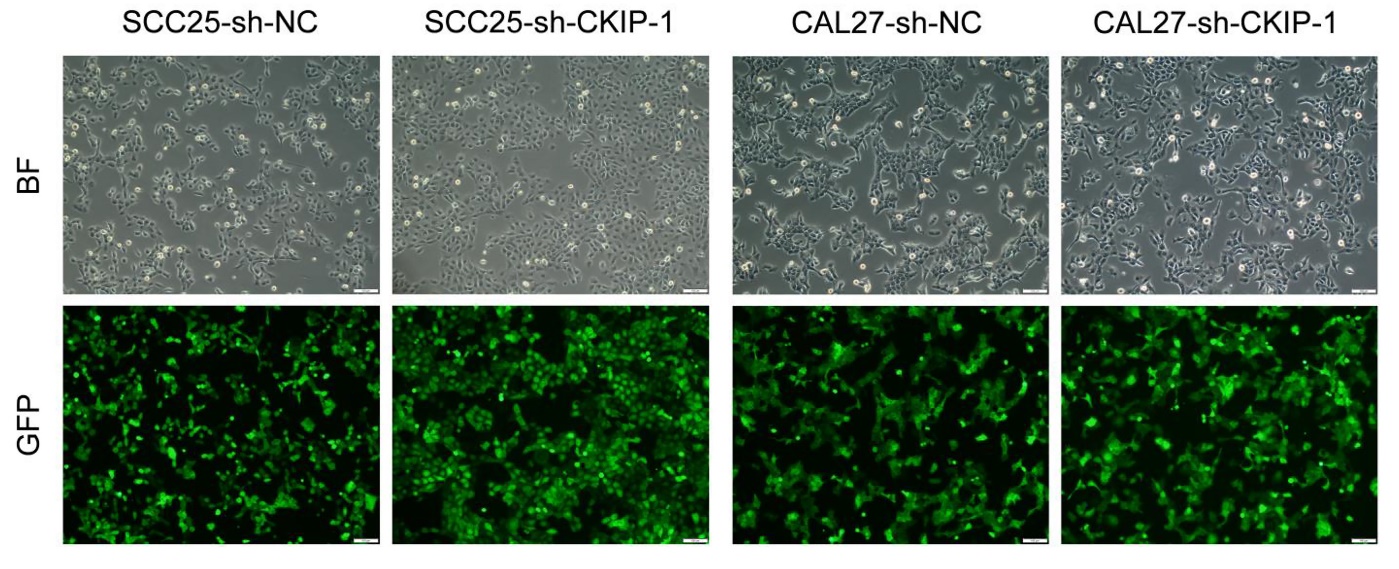


**Figure S3:** The stable knockdown efficiency of CKIP-1 in OSCC cells was verified by fluorescence imaging.


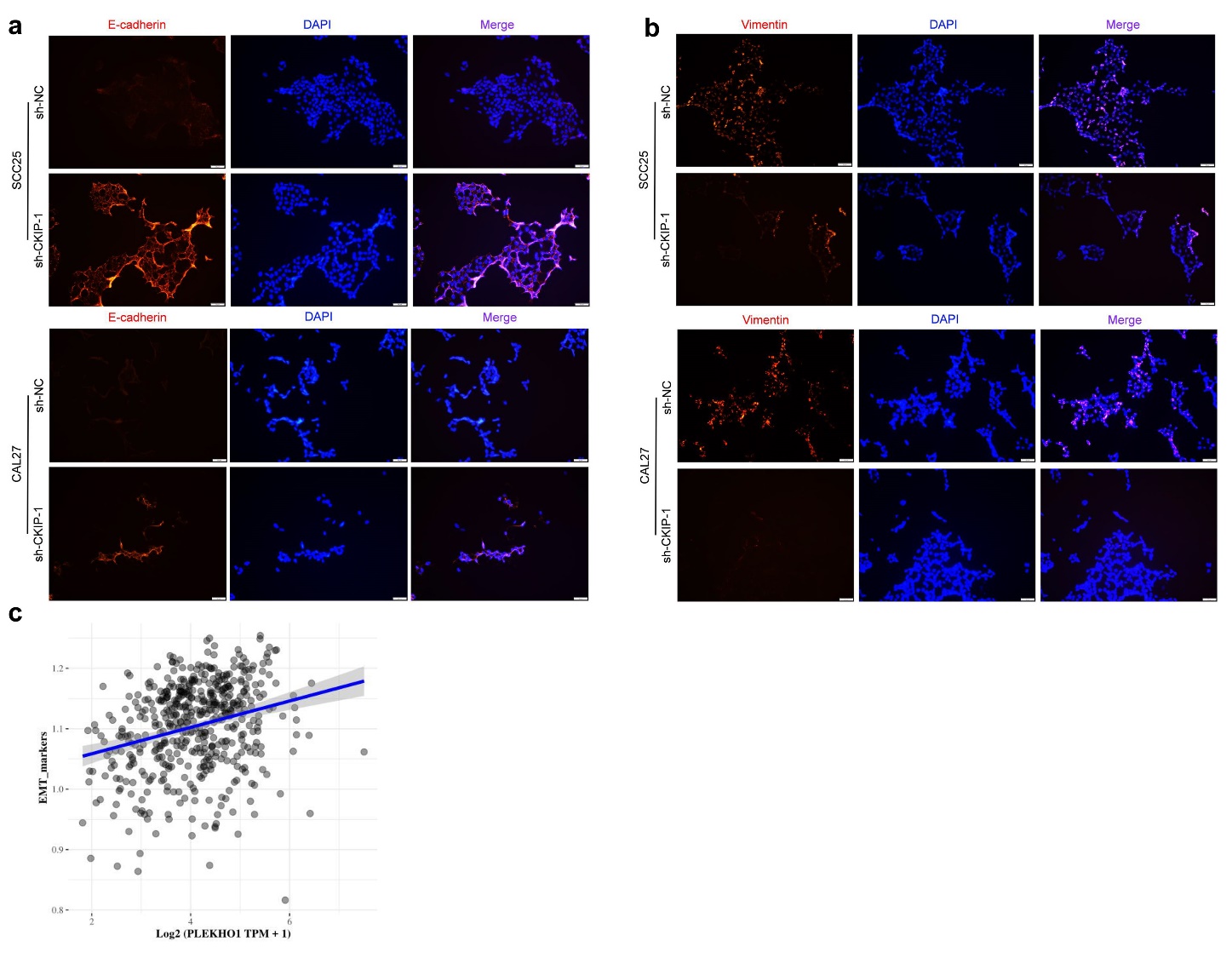


**Figure S4:** Immunofluorescence staining showed that knockdown of CKIP-1 increased E-cadherin expression **(a)** and decreased Vimentin expression **(b)** in OSCC cells. Pathway analysis of the public database showed that CKIP-1 expression was positively correlated with EMT **(c**). Scale bar: 50 μm.


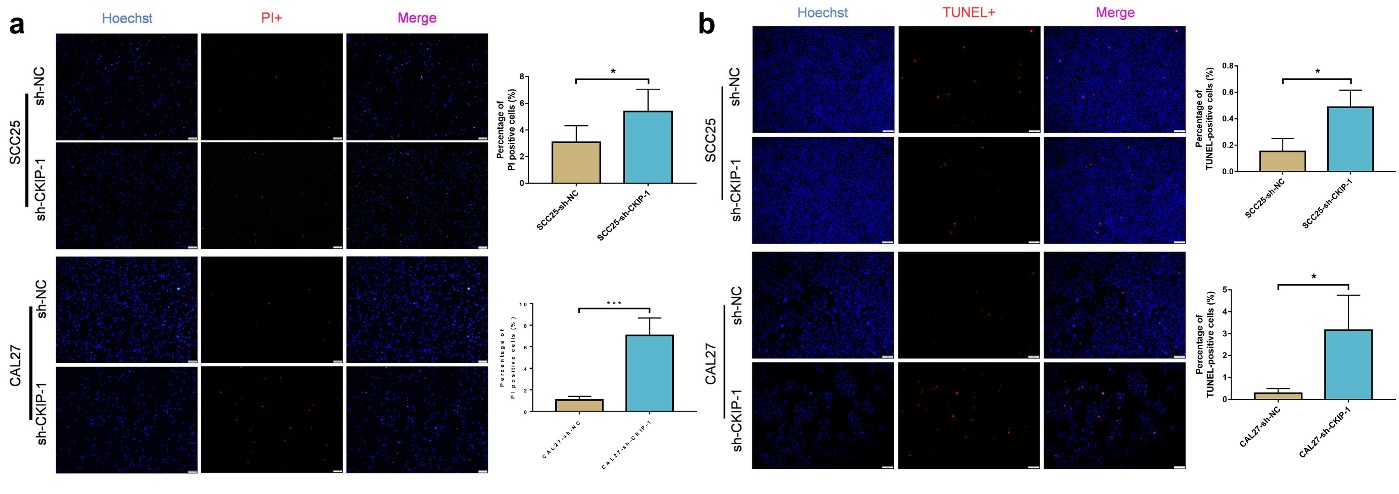


**Figure S5:** TUNEL staining **(a)** and PI staining **(b)** showed that the proportion of TUNEL positive and PI positive cells increased when OSCC cells were knocked down with CKIP-1. Significance was defined as **P* < 0.05 and ****P* < 0.001. Scale bar: 100 μm.

**
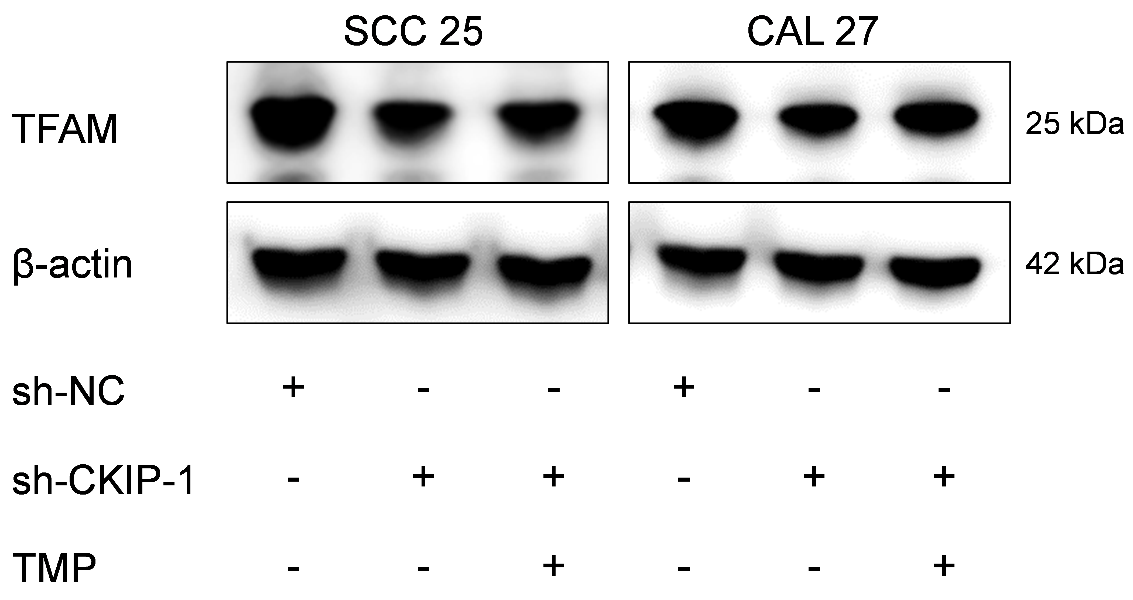
**

**Figure S6:** The inhibition of CKIP-1 knockdown-caused TFAM degradation by TMP in OSCC cells was detected by Western blotting.

**Table S1**: Small interfering RNAs

| siRNA | The sequence of the siRNA |
| --- | --- |
| si-CKIP-1-homo-917 | sense, 5’-GGACUUGAUCCAAGAGGAATT-3’  antisense, 5’-UUCCUCUUGGAUCAAGUCCTT-3’) |
| si-CKIP-1-homo-1178 | sense, 5’-GAUCCUAUCUCAGCGGGAUTT-3’  antisense, 5’- AUCCCGCUGAGAUAGGAUCTT-3’ |
| si-CKIP-1-homo-1434 | sense, 5’-GGAGAGGCAUCAUCGAAUUTT-3’  antisense, 5’-AAUUCGAUGAUGCCUCUCCTT-3’ |
| si-NC | sense, 5’-UUCUCCGAACGUGUCACGUTT-3’  antisense, 5’-ACGUGACACGUUCGGAGAATT-3’ |

**Table S2**: Primers used for RT-qPCR (Sangon, Shanghai, China)

| Gene | The sequence of the primers (5’-3’) |
| --- | --- |
| *CKIP-1* | F-AATTCTGCGGGAAAGGGATTT  R-AACACCTCCTGACTGTTTTCTC |
| *GAPDH* | F-CTTTGGTATCGTGGAAGGACTC  R-CAGTAGAGGCAGGGATGATGTT |

F, forward; R, reverse; CKIP-1, casein kinase-2 interaction protein-1; GAPDH, glyceraldehyde-3-phosphate dehydrogenase.

**Table S3**: Antibodies for WB, IF and IHC

| Name | Brand | Catalog number | Dilution rate |
| --- | --- | --- | --- |
| Bax | Abcam, Cambridge, UK  Abcam, Cambridge, UK | ab32503 | 1:5000 (WB) |
| Bcl2 |  | ab32124 | 1:1000 (WB) |
| CKIP-1 | Santa Cruz, TX, USA | sc-376355 | 1:200 (WB), 1:50 (IF, IHC) |
| Caspase 3 | Abcam, Cambridge, UK | ab32351 | 1:1000 (WB) |
| cGAS | Abcam, Cambridge, UK | ab252416 | 1:1000 (WB) |
| E-cadherin | CST, Danvers, USA | #3195 | 1:1000 (WB), 1:500 (IF) |
| Ki67 | Abmart, Shanghai, China | TW0001 | 1:200 (IF) |
| Vimentin | Abcam, Cambridge, UK | ab9254 | 1:1000 (WB), 1:200 (IF) |
| TFAM | Proteintech, CA, USA | 22586-1-AP | 1:1000 (WB), 1:200 (IF) |
| STING | Abcam, Cambridge, UK | ab239074 | 1:1000 (WB) |
| β-actin | Proteintech, CA, USA | 66009-1-Ig | 1:20000 (WB) |

WB: Western blotting; IHC: immunohistochemistry; IF: immunofluorescence
